# Cesena guidelines: WSES consensus statement on laparoscopic-first approach to general surgery emergencies and abdominal trauma

**DOI:** 10.1186/s13017-023-00520-9

**Published:** 2023-12-08

**Authors:** Giacomo Sermonesi, Brian W. C. A. Tian, Carlo Vallicelli, Fikri M. Abu‑Zidan, Dimitris Damaskos, Michael Denis Kelly, Ari Leppäniemi, Joseph M. Galante, Edward Tan, Andrew W. Kirkpatrick, Vladimir Khokha, Oreste Marco Romeo, Mircea Chirica, Manos Pikoulis, Andrey Litvin, Vishal Girishchandra Shelat, Boris Sakakushev, Imtiaz Wani, Ibrahima Sall, Paola Fugazzola, Enrico Cicuttin, Adriana Toro, Francesco Amico, Francesca Dal Mas, Belinda De Simone, Michael Sugrue, Luigi Bonavina, Giampiero Campanelli, Paolo Carcoforo, Lorenzo Cobianchi, Federico Coccolini, Massimo Chiarugi, Isidoro Di Carlo, Salomone Di Saverio, Mauro Podda, Michele Pisano, Massimo Sartelli, Mario Testini, Andreas Fette, Sandro Rizoli, Edoardo Picetti, Dieter Weber, Rifat Latifi, Yoram Kluger, Zsolt Janos Balogh, Walter Biffl, Hans Jeekel, Ian Civil, Andreas Hecker, Luca Ansaloni, Francesca Bravi, Vanni Agnoletti, Solomon Gurmu Beka, Ernest Eugene Moore, Fausto Catena

**Affiliations:** 1 Department of General and Emergency Surgery, Bufalini Hospital‐Level 1 Trauma Center, Cesena, Italy; 2 Department of General Surgery, Singapore General Hospital, Singapore, Singapore; 3 Department of Surgery, College of Medicine and Health Sciences, United Arab Emirates University, Al‑Ain, United Arab Emirates; 4 Department of Surgery, Royal Infirmary of Edinburgh, Edinburgh, UK; 5 Department of General Surgery, Albury Hospital, Albury, Australia; 6 Abdominal Center, Helsinki University Hospital and University of Helsinki, Helsinki, Finland; 7 Division of Trauma and Acute Care Surgery, Department of Surgery, University of California Davis, Sacramento, CA USA; 8 Department of Surgery, Radboud University Medical Center, Nijmegen, The Netherlands; 9 Departments of Surgery and Critical Care Medicine, University of Calgary, Foothills Medical Centre, Calgary, AB Canada; 10 Department of Emergency Surgery, City Hospital, Mozyr, Belarus; 11 Trauma, Burn, and Surgical Care Program, Bronson Methodist Hospital, Kalamazoo, MI USA; 12 Department of Digestive Surgery, Centre Hospitalier Universitaire Grenoble Alpes, La Tronche, France; 13 3Rd Department of Surgery, Attikon General Hospital, National and Kapodistrian University of Athens (NKUA), Athens, Greece; 14 Department of Surgical Diseases No. 3, Gomel State Medical University, Gomel, Belarus; 15 Department of General Surgery, Tan Tock Seng Hospital, Novena, Singapore; 16 General Surgery Department, Medical University, University Hospital St George, Plovdiv, Bulgaria; 17 Department of Surgery, Sheri-Kashmir Institute of Medical Sciences, Srinagar, India; 18 General Surgery Department, Military Teaching Hospital, Dakar, Senegal; 19 Department of Surgery, Fondazione IRCCS Policlinico San Matteo, University of Pavia, Pavia, Italy; 20 Department of General, Emergency and Trauma Surgery, Pisa University Hospital, Pisa, Italy; 21 Department of Surgical Sciences and Advanced Technologies, General Surgery Cannizzaro Hospital, University of Catania, Catania, Italy; 22 Discipline of Surgery, School of Medicine and Public Health, Newcastle, Australia; 23 Department of Management, Ca’ Foscari University of Venice, Campus Economico San Giobbe Cannaregio, 873, 30100 Venice, Italy; 24 Department of Emergency Surgery, Centre Hospitalier Intercommunal de Villeneuve-Saint-Georges, Villeneuve-Saint-Georges, France; 25 Donegal Clinical Research Academy Emergency Surgery Outcome Project, Letterkenny University Hospital, Donegal, Ireland; 26 Department of Surgery, IRCCS Policlinico San Donato, University of Milano, Milan, Italy; 27 Department of Surgical Science, Istituto Clinico Sant’Ambrogio, Milan, Italy; 28 Department of Surgery, S. Anna University Hospital and University of Ferrara, Ferrara, Italy; 29 General Surgery Department Hospital of San Benedetto del Tronto, Marche Region, Italy; 30 Department of Surgical Science, Emergency Surgery Unit, University of Cagliari, Cagliari, Italy; 31 General and Emergency Surgery, ASST Papa Giovanni XXIII, Bergamo, Italy; 32 Department of Surgery, Macerata Hospital, Macerata, Italy; 33 Department of Precision and Regenerative Medicine and Ionian Area, Unit of Academic General Surgery, University of Bari “A. Moro”, Bari, Italy; 34 Pediatric Surgery, Children’s Care Center, SRH Klinikum Suhl, Suhl, Thuringia Germany; 35 Surgery Department, Section of Trauma Surgery, Hamad General Hospital (HGH), Doha, Qatar; 36 Department of Anesthesia and Intensive Care, Azienda Ospedaliero‑Universitaria Parma, Parma, Italy; 37 Department of Trauma Surgery, Royal Perth Hospital, Perth, Australia; 38 Department of Surgery, Westchester Medical Center, New York Medical College, Valhalla, NY USA; 39 Department of General Surgery, Rambam Health Care Campus, Haifa, Israel; 40 Department of Traumatology, John Hunter Hospital and University of Newcastle, Newcastle, NSW Australia; 41 Division of Trauma/Acute Care Surgery, Scripps Clinic Medical Group, La Jolla, CA USA; 42 Department of Surgery, Erasmus University Medical Centre, Rotterdam, The Netherlands; 43 Faculty of Medical and Health Sciences, University of Auckland, Auckland, New Zealand; 44 Emergency Medicine Department of General and Thoracic Surgery, University Hospital of Giessen, Giessen, Germany; 45 Healthcare Administration, Santa Maria Delle Croci Hospital, Ravenna, Italy; 46 Ethiopian Air Force Hospital, Bishoftu, Oromia Ethiopia; 47 Ernest E Moore Shock Trauma Center at Denver Health, University of Colorado, Denver, CO USA

**Keywords:** Laparoscopy, Laparoscopic approach, Minimally invasive surgery/approach, Emergency general surgery, Acute care surgery, Trauma surgery hemodynamic stability, Acute peritonitis, Acute appendicitis, Acute cholecystitis, Incarcerated/complicated ventral/inguinal hernia, Adhesive small bowel obstruction, Acute diverticulitis, Colo–rectal emergencies, Mesenteric ischemia, Perforated peptic ulcer, Acute pancreatitis, Penetrating/blunt abdominal trauma, Guidelines, Recommendations

## Abstract

**Background:**

Laparoscopy is widely adopted across nearly all surgical subspecialties in the elective setting. Initially finding indication in minor abdominal emergencies, it has gradually become the standard approach in the majority of elective general surgery procedures. Despite many technological advances and increasing acceptance, the laparoscopic approach remains underutilized in emergency general surgery and in abdominal trauma. Emergency laparotomy continues to carry a high morbidity and mortality. In recent years, there has been a growing interest from emergency and trauma surgeons in adopting minimally invasive surgery approaches in the acute surgical setting. The present position paper, supported by the World Society of Emergency Surgery (WSES), aims to provide a review of the literature to reach a consensus on the indications and benefits of a laparoscopic-first approach in patients requiring emergency abdominal surgery for general surgery emergencies or abdominal trauma.

**Methods:**

This position paper was developed according to the WSES methodology. A steering committee performed the literature review and drafted the position paper. An international panel of 54 experts then critically revised the manuscript and discussed it in detail, to develop a consensus on a position statement.

**Results:**

A total of 323 studies (systematic review and meta-analysis, randomized clinical trial, retrospective comparative cohort studies, case series) have been selected from an initial pool of 7409 studies. Evidence demonstrates several benefits of the laparoscopic approach in stable patients undergoing emergency abdominal surgery for general surgical emergencies or abdominal trauma. The selection of a stable patient seems to be of paramount importance for a safe adoption of a laparoscopic approach. In hemodynamically stable patients, the laparoscopic approach was found to be safe, feasible and effective as a therapeutic tool or helpful to identify further management steps and needs, resulting in improved outcomes, regardless of conversion. Appropriate patient selection, surgeon experience and rigorous minimally invasive surgical training, remain crucial factors to increase the adoption of laparoscopy in emergency general surgery and abdominal trauma.

**Conclusions:**

The WSES expert panel suggests laparoscopy as the first approach for stable patients undergoing emergency abdominal surgery for general surgery emergencies and abdominal trauma.

## Introduction and background

Laparoscopy is a widely adopted minimally invasive surgical technique. Initially used in emergency minor surgery (e.g. appendectomy), laparoscopy has progressively gained favor due to its improved outcomes, and it is now becoming the standard approach in the majority of elective general surgery procedures.

Despite growing evidence of the potential benefit of the laparoscopic approach in a variety of emergency settings, its actual adoption remains low in practice. The results of a recent research study from the National Emergency Laparotomy Audit (NELA) of England and Wales described that only 14.6% of cases were approached by laparoscopy with a conversion rate of 46.4% [1]. A research study from the USA reported a higher proportion (69.4%) of minimally invasive surgery (MIS) in emergency general surgery, but the majority of interventions were appendectomy and cholecystectomy: the proportion of other emergency abdominal surgery procedures performed with MIS was less than 20% [2].

A WSES survey conducted amongst 415 surgeons from 67 different countries, lately confirms that laparoscopy is used in less than 20% of major emergency operations [3]. The strongest deterrent to the use of MIS in emergency surgery was the patient’s poor physiological condition. Other important limiting factors include previous abdominal surgery and estimated prolonged surgical duration. Conversely, when laparoscopy was attempted, the main reasons for conversion were the deterioration of clinical conditions, unclear anatomical visualization, bowel perforation and bleeding. Surgeons expressed confidence in MIS techniques for relatively simple emergencies such as appendicectomy, cholecystectomy or abdominal exploration. This confidence progressively decreased with the increasing complexity of the surgical procedure. Surgeons’ exposure to laparoscopy in elective surgeries and increased surgical experience were factors leading to an increased adoption of MIS in the emergency setting. Emergency and trauma surgery practice usually requires dedicated teams with specific skills and competences [4, 5] that may not include MIS techniques.

The learning process and skills required for elective MIS techniques are largely documented in the literature [6–9]. Conversely, the corresponding MIS training process in emergency surgery has been rarely investigated. This is probably due to a lack of established benchmarks, standards and goals in this field. In order to stimulate discussion and further research, a WSES position paper on a Training Curriculum in minimally invasive emergency digestive surgery was recently published [10].

A recent analysis in the USA found that 20% of the hospitalized population undergoes trauma or emergency general surgery procedures, accounting for 25% of inpatient costs [11]. Moreover, the emergency general surgery population is likely to be elderly, with prolonged length of stay, worse outcomes and a bimodal distribution of death. All these factors contribute towards a major impact on healthcare utilization rates [11–13]. In the absence of hemorrhagic/septic shock and signs of severe physiological derangement, the appropriate surgical approach in emergency abdominal surgery remains unclear. Emergency laparotomy remains mandatory for unstable patients, but studies have reported that patients undergoing emergency laparotomy carry the highest- morbidity and mortality rates [2, 14, 15]. In recent years, a growing body of evidence has shown favorable outcomes with the adoption of a laparoscopic approach, when feasible, in emergency general surgery and abdominal trauma patients. This suggests that there is an opportunity to improve the clinical results of the acute care surgery and trauma population by encouraging MIS in appropriate cases.

The present position paper, endorsed by the World Society of Emergency Surgery (WSES), aims to provide a review of the literature to reach a consensus on the indications and benefits of a laparoscopic-first approach in patients requiring emergency abdominal surgery.

### Project rationale and design

The present position paper, conducted according to the WSES methodology [16], aims to provide a review of the literature investigating the use of the laparoscopic approach in emergency general surgery and abdominal trauma patients meeting the indications, to develop a shared consensus statement based on the currently available evidence.

Two authors (GS and BT) performed the literature review and subsequently coordinated with the panel of international experts to draft the present position paper. The international panel included 54 experts who were asked to critically revise and discuss the manuscript to develop the position statement. The final grade of the statement was assessed according to the Grades of Recommendation, Assessment, Development, and Evaluation (GRADE) system [17].

### Purpose and use of these guidelines

These guidelines are evidence-based, with the grades of recommendation based on the evidence and a consensus of experts. They do not exclude other approaches as being within a standard of practice. The treating clinician should determine the most appropriate action, after taking into account conditions at the relevant medical institution (staff levels, experience, equipment, etc.) and the characteristics of the individual patient. The responsibility for the management and outcome rests with the engaging practitioners, and not the consensus group.

## Methods

### Review question, selection criteria, and search strategy

A review of the literature was performed and reported according to the Preferred Reporting Items for Systematic Reviews and Meta-Analyses (PRISMA) statement [18].

Studies on laparoscopic approaches in the management of abdominal surgical emergencies including general surgery emergencies and abdominal trauma were retrieved from the following databases on May 1, 2023: MEDLINE (through PubMed), Embase, and the Cochrane Library.

The focus question was the following: is laparoscopy suggested as the first approach for stable patients requiring emergency abdominal surgery for general surgery emergencies or abdominal trauma?

A specific research query was formulated for each database, using the following keywords and MeSH terms: laparoscopy, laparoscopic, laparoscopic approach, minimally invasive surgery/approach, emergency general surgery, acute care surgery, hemodynamically stable, hemodynamic stability, acute peritonitis, acute appendicitis, acute cholecystitis, incarcerated/complicated ventral/inguinal hernia, adhesive small bowel obstruction, acute diverticulitis, colo–rectal emergencies, acute mesenteric ischemia, perforated peptic ulcer, severe acute pancreatitis, penetrating abdominal trauma, blunt abdominal trauma. Terms were variously combined, with the use of the Boolean operators “AND” and “OR.”

According to the PICOS format, the following items were used as selection criteria for articles emerging from the literature search: P, Population: adult patients with general surgery emergencies or abdominal trauma requiring emergency surgery. I**,** Intervention: laparoscopic approach and minimally invasive procedures C, Comparisons: open surgery or no comparison**.** O, outcome(s): operative and postoperative outcomes S, Study design: clinical trials, consensus conferences, comparative studies, guidelines, government publications, multicenter studies, systematic reviews, meta-analyses, randomized controlled trials, large case series, original articles were included.

Two reviewers (GS and BT) screened the list of articles. All records were reviewed for relevance concerning the title and abstract. Records were removed when both reviewers excluded them. Otherwise, the disagreement was resolved via a discussion/ intervention of a tiebreaker (FC). Both reviewers then performed an independent full-text analysis, which allowed them to finally include or exclude the preselected article.

### Data extraction and synthesis

Data extraction was performed by filling in an electronic spreadsheet, which included the following items: first author’s name, year of publication, scientific journal, type of study (or study design), number of patients included, disease requiring surgical intervention, type of surgical intervention, surgical approach, operative and postoperative surgical outcomes, cost analysis data when available. The risk of bias in the selected studies was assessed by using validated systems according to the study design [19–21].

### Quality assessment and analysis

To maintain the quality of the review, abstracts of the articles were checked for evaluation and analysis of the articles to ensure the quality and relevance of the literature included in the review. Due to the heterogeneity of selected studies, we performed a qualitative analysis because a quantitative analysis would be considered as inappropriate. Evidence synthesis was also done based on the validity of the method used, novelty and clarity of results.

## Results

### Literature search and selection

**Fig. 1:**
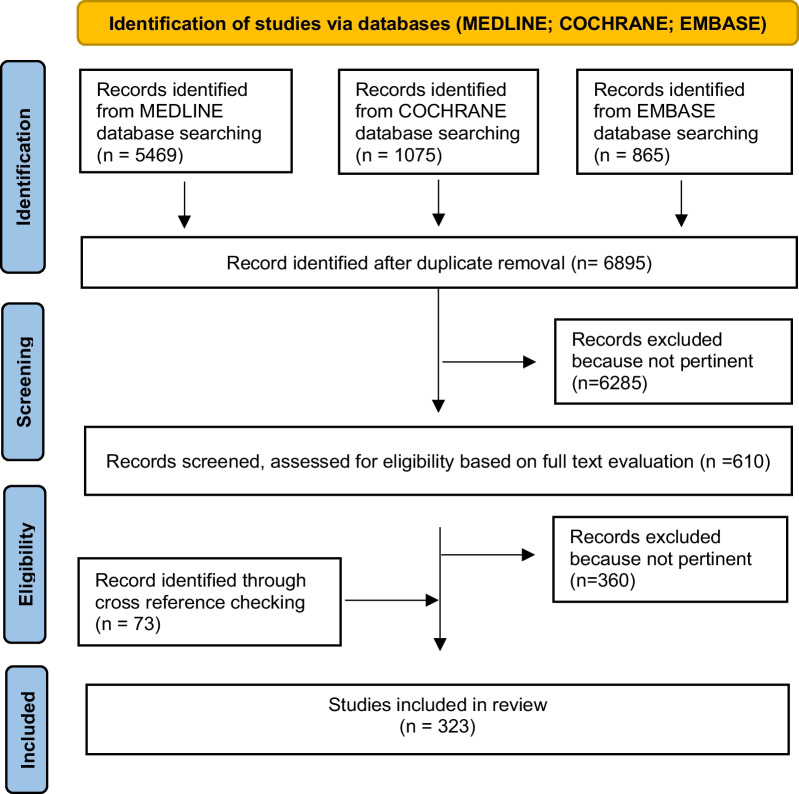
Shows PRISMA flow chart of Review paper

The initial search yielded 7409 results (PubMed 5469, Cochrane 1075, Embase 865). After removing duplicates, 6895 articles were screened for eligibility based on title and abstract, and 610 articles were retrieved for a full-text evaluation. After excluding 360 non pertinent articles, a total of 323 studies were finally included in the review, including 73 articles identified through cross reference checking (Fig. 1).

### Overview of laparoscopy in emergency abdominal surgery

In patients requiring emergency abdominal surgery for general surgery emergencies or abdominal trauma, it is crucial to identify parameters to assess the severity of disease (e.g. to establish if a patient is stable or unstable).

The following criteria have been reported in adult patients [22–24]:

- normal hemodynamic status: the patient does not require fluids or transfusions to maintain blood pressure, without signs of hypoperfusion;
- hemodynamic stability: the patient has a systolic blood pressure > 90 mmHg (or a mean arterial blood pressure > 65 mmHg) and a heart rate < 100 bpm and base excess (BE) > -5 mmol/l after intravenous fluid;
- hemodynamic instability: the patient has an admission systolic blood pressure < 90 mmHg (or a mean arterial blood pressure < 65 mmHg) with clinical evidence of hemorrhagic shock [skin vasoconstriction (cool, clammy, decreased capillary refill), altered level of consciousness and/or shortness of breath], or an admission systolic blood pressure > 90 mmHg (or a mean arterial blood pressure > 65 mmHg) but requiring intravenous fluid/transfusions or vasopressor drugs and/or an admission base excess (BE) > − 5 mmol/l and/or shock index > 1 and/or transfusion requirement of at least > 4 units of packed red blood cells within the first 8 h; the transient responder patients are those showing an initial response to adequate fluid resuscitation, but subsequently developing signs of ongoing blood loss recurring instability and/or perfusion deficits. Transient responder patients should be considered as hemodynamically unstable.

These criteria for the definition of the hemodynamic state have been published and variably validated for the abdominal injured trauma patients. As general surgery patients with severe intraperitoneal sepsis or bleeding are just as susceptible to the detrimental effects of acidosis, hypothermia and coagulopathy, these criteria were subsequently adopted for the definition of the patient’s hemodynamic state in non-traumatic abdominal emergencies [25–27]. For this purpose, some authors have proposed other criteria to establish patient’s instability [27–29]: T(°C) < 35, pH < 7·20, Lactate > 2.5 (the lethal triad); systolic blood pressure < 70 mmHg; base deficiency >  − 8.0 [6]; INR > 1.7; need for the introduction /titration of intraoperative norepinephrine > 10 mcg/min.

Instability is mostly established by parameters (systolic blood pressure, mean arterial blood pressure, heart rate, temperature—hypothermia), physical examination (vasoconstriction, oliguria, decreased level of consciousness) laboratory evidence of tissue hypoperfusion (pH, lactate/base deficit, coagulopathy). However, there are no universally accepted definitions for the classification of the patient’s hemodynamic status and clinical scenarios are often complicated. What may be regarded as acceptable physiological parameters will vary depending on many factors including the age, underlying medication and comorbidities of the patient. Therefore, blood pressure goals should be individualized by the physician according to patient physiology, comorbidities and physiological compensation to shock during the time of resuscitation [30].

For unstable patients suffering from septic shock due to peritonitis, or hemorrhagic shock due to abdominal bleeding, an open laparotomy as first approach remains mandatory. In a review on Emergency Laparoscopy published in the World Journal of Emergency Surgery in 2006, Warren et al. summarized the indications for emergency laparoscopy based on the contemporary evidence [31]. Laparoscopic surgery was established as the best intervention in acute appendicitis, acute cholecystitis and most gynecological emergencies. In penetrating thoraco-abdominal trauma stable patients, where computed tomography (CT) scan has the low sensitivity in detecting diaphragmatic injury, a laparoscopic approach was found to be effective not only for the diagnosis but also as a treatment option. However, its role in the management of other more technically demanding general surgical emergencies, such as perforating peptic ulcers, acute mesenteric ischemia, acute diverticulitis, incarcerated hernias, small bowel obstructions, as well as the majority of abdominal trauma requiring surgery, was considered unclear and the evidence was insufficient to justify the adoption of a laparoscopic-first approach.

More recent reviews have occasionally widened the indications for the laparoscopic approach to include other abdominal emergencies requiring surgery (e.g. perforated peptic ulcer), in which, however, its utility and advantages were mostly considered under debate or not significant [32–34]. Some reviews have focused only on the field of abdominal emergencies or abdominal trauma separately considering for example the presence of peritonitis as a contraindication to the laparoscopic approach in abdominal trauma [35, 36].

### Laparoscopic approach in acute appendicitis (AA)

The 2020 update of the WSES Jerusalem guidelines recommends laparoscopic appendectomy as the preferred approach over open appendectomy, for both uncomplicated and complicated acute appendicitis (AA) [37].

At the beginning of the last decade, two systematic reviews of randomized controlled trials (RCTs) comparing laparoscopic appendectomy (LA) versus open appendectomy (OA), reported less postoperative pain and less postoperative complications, lower surgical site infections (SSI) and shorter length of stay (LOS), in patients undergoing a laparoscopic approach [38, 39]. In a review of 9 systematic reviews of RCTs, Jaschinski et al. [40], found a pooled duration of surgery relatively longer (7.6 to 18.3 min) with laparoscopy. Whereas the occurrence of SSI pooled by all reviews was lower after LA, in half of six meta-analyses, the risk of intra-abdominal abscesses (IAA) was higher, as also noted in the 2018 updated Cochrane review on laparoscopic versus open surgery for suspected appendicitis [41].

Nevertheless, the evidence regarding the treatment effectiveness of LA versus OA in terms of postoperative IAA changed over past decades. The cumulative meta-analysis by Ukai et al. demonstrated that, of the 51 trials addressing IAA, trials published up to 2001, showed statistical significance in favor of OA, but the effect size began to disappear after 2001 [42]. Indeed, no significant difference in IAA rates was found by Athanasiou et al. in a meta-analysis on studies from 1999 onwards comparing LA and OA for complicated AA. LA appears to have significant benefits with improved morbidity, significantly less SSI, reduced time to oral intake and LOS. Operative time was longer during LA without reaching statistical significance in the RCT subgroup analysis [43]. The benefit of LA over OA was confirmed in complicated AA by other SRs on perforated appendicitis [44] and from a multicenter cohort study in diffuse peritonitis from perforated AA [45]. A shorter postoperative hospital stays and fewer SSI was found in LA groups and no significant difference were found in terms of intra-abdominal abscess, postoperative peritonitis, rate of reoperation, and mortality.

Moreover Zhang et al. [46] found shorter operative time in the LA group in a recent meta-analysis of 9 RCTs and 7 retrospective studies published between 2010 and 2021, suggesting that the evidence regarding the surgical time of laparoscopic appendectomy has also been changing over the last decade, with the increase of laparoscopic surgical skills and experience in performing LA.

Appendicitis in elderly patients is associated with an increased risk of postoperative complications. In a systematic review of twelve studies, Wang et al. 2019, showed that LA is safe and feasible in elderly patients with AA and results in lower mortality, postoperative morbidity and shorter hospitalization when compared with OA [47].

Few large-scale epidemiologic studies evaluate the clinical and economic burden of appendicitis [48–50]. Masoomi et al. published the largest population-based study examining a total of 2,593,786 patients who underwent appendectomy for acute appendicitis from 2004 to 2011. The utilization of LA significantly increased from 43.3% in 2004 to 75% in 2011. Compared with OA, LA had a significantly lower complication rate, a lower mortality rate, a shorter mean hospital stays, and lower mean total hospital charges in both nonperforated and perforated appendices [50].

### Laparoscopic approach in acute calculus cholecystitis (ACC)

The 2020 World Society of Emergency Surgery updated guidelines for the diagnosis and treatment of acute calculous cholecystitis (ACC) recommended laparoscopic cholecystectomy (LC) as the first-line treatment for patients with ACC. Septic shock or anesthesia-related contraindications are reasons to avoid a laparoscopic approach [51].

In the past decades, acute cholecystitis was considered an absolute contraindication to laparoscopy. However, over the years, the growing evidence of LC feasibility and safety in ACC, together with the increase in surgical laparoscopic experience and equipment, has resulted in low utilization of the open approach.

Nevertheless, a 2015 worldwide survey on intra-abdominal infection, the CIAOW study [52] showed that half of patients with ACC still underwent open surgery, despite only 14% of patients enrolled in the study being in critical condition prior to surgery (severe sepsis or septic shock). The CIAOW study’s findings were disappointing as the uptake of a laparoscopic-first approach was low, despite the growing evidence from systematic reviews and meta-analyses published in the same year which compared the outcomes of open versus laparoscopic cholecystectomy in ACC. Laparoscopy was found to reduce post-operative mortality, morbidity, pneumonia rate, wound infection rates and LOS. Intraoperative blood loss, bile leakage and operative time were not influenced by the approach. The difference in the mean operative time was progressively in favor of laparoscopy from 1998 to 2007 [53].

The laparoscopic approach to ACC in emergency settings, improves not only the patients’ outcomes, but also improves the utilization of health care resources. Early LC was associated with a significant reduction in wound infection rate, hospitalization length, duration of surgery and quality of life, compared to delayed LC as demonstrated by Song et al. in a meta-review of seven discordant meta-analyses and systematic reviews published from 2004 to 2015. No differences were found in mortality, bile duct injury, bile leakage, overall complications and conversion to open surgery [54]. Early LC demonstrated its superiority also in terms of cost-effectiveness, as demonstrated in a meta-analysis on six studies containing cost analyses that compared early versus delayed LC for ACC [55].

The Tokyo Guidelines 18 (TG18) widened the indications for LC when compared with Tokyo Guidelines 13 (TG13), as they supported same-admission LC for patients with all three severity grades of ACC [56, 57]. A recent meta‑analysis and systematic review confirmed the superiority of emergency cholecystectomy over percutaneous cholecystostomy for the treatment of ACC also in high‑risk surgical patients, in terms of mortality/morbidity, readmission rate, and LOS [58].

The risk of a laparoscopic approach failure and the need for conversion increases in the case of a difficult gallbladder. The incidence of difficult cholecystitis reported in the literature is 10–15% of the total cases of ACC [59]. Major factors that contribute towards a difficult cholecystectomy include the severity of the disease, the presence of adhesions, the surgeon’s laparoscopic experience including caseload of operations for ACC, and the devices availability for surgical treatment [60, 61]. Conversion rates were increased in the presence of previous upper midline abdominal surgery, as shown in a recent prospective study [62] and in advanced ACC with high CRP, gangrene or an abscess [63].

A review conducted in 2011 showed no consensus on the ideal way to deal with a difficult gallbladder. The options include subtotal cholecystectomy, fundus first cholecystectomy, intra-operative cholangiogram, open conversion or a combination of these options [64]. Due to the diversity of reasons and the variability of approaches among surgeons, no consensus has been reached regarding the choice of the most appropriate “bailout technique” [65, 66]. The 2020 WSES guidelines recommended conversion from laparoscopic to open cholecystectomy in case of severe local inflammation, adhesions, bleeding within Calot’s triangle or suspected bile duct injury [51]. However, both bile duct injury and conversions significantly increase morbidity and mortality, adversely affecting the quality of life while being associated with substantial costs [67–69].

Furthermore, in the laparoscopic era, where junior surgeons and trainees have had limited exposure to open cholecystectomy [70], the development of effective alternative minimally invasive strategies that may allow avoidance of conversion, would be optimal. Difficult gallbladders can be managed through various laparoscopic techniques such as fundus-first laparoscopic cholecystectomy or laparoscopic reconstituting subtotal cholecystectomy allowing reduced conversion rates and associated morbidity [71–73]. A recent systematic review had supported the use of percutaneous drainage as a bridge to surgery in high- risk ACC, especially in patients with higher perioperative risks or longstanding ACC in an effort to reduce biliary leakage and postoperative complications [74]. Appropriate patient selection for surgery still represents a source of debate. A recent prospective multicenter observational study, the S.P.Ri.M.A.C.C. study, found the CHOLE-POSSUM as a reliable tool to stratify patients with ACC into a low-risk group that may represent safe early cholecystectomy candidates, and a high-risk group, where new minimally invasive techniques may be the most useful course of action [75].

In a recent systematic review and meta-analysis comparing endoscopic ultrasound guided gallbladder drainage (EUS-GBD) versus percutaneous gallbladder drainage (PT-GBD) for patients with ACC who were unfit for surgery [76], Hemerly et al. found that EUS-GBD using cautery-enhanced lumen apposing metal stent (LAMS) was superior to PT-GBD in terms of safety profile, recurrent cholecystitis, and hospital readmission rates.

Indocyanine Green (ICG) fluorescence is increasingly integrated in the laparoscopic armamentarium, and a recent meta-analysis has shown its safety and effectiveness in improving the visualization of the extrahepatic biliary tree during LC, as compared to intraoperative cholangiography [77]. However, these studies were mostly performed in elective settings. In a first series looking at the use of indocyanine green in the acute care surgery population, the operative time or need for a bail-out operation were not decreased [78]. However, as with most retrospective studies, the conclusions achieved by the authors are questionable and considering the rising importance of ICG in hepatobiliary surgery [79], further prospective studies are needed to assess its potential use in emergency settings.

### Laparoscopic approach in complicated Groin and Ventral Hernia

According to the 2017 update of the WSES guidelines for emergency repair of complicated abdominal wall hernias, the repair of incarcerated hernias—both ventral and groin—may be performed with a laparoscopic approach in the absence of strangulation and suspicion of the need for bowel resection, where an open approach is preferable [80]. However, due to improvements in MIS techniques and equipment, the laparoscopic approach is increasingly chosen for acute incarcerated hernias.

#### Groin Hernia

An important advancement of groin hernia repair was the introduction of MIS, which provides earlier return to daily activities, lower postoperative pain, reduced need for analgesics and lower incidence of wound infection in comparison to the open approach [81–83]. In addition, a recent meta-analysis has found a lower incidence of chronic groin pain following laparoscopic repair, with no differences in recurrence rate compared to open repairs [84].

The laparoscopic approach for the treatment of incarcerated groin hernias is still debated, especially in the emergency setting [85–87]. The 2017 WSES guidelines on emergency repair of complicated abdominal wall hernias [86] recommended using a laparoscopic approach only with the aim to assess bowel perfusion after spontaneous reduction of strangulated hernia. On the other hand, Deeba et al. in 2009, in a systematic review focused on the laparoscopic treatment of acutely incarcerated inguinal hernias [87] showed that a laparoscopic approach, with both transabdominal preperitoneal (TAPP) or totally extraperitoneal (TEP) repair, was safe and feasible not only for chronic incarcerated hernias. The overall rate of complication, recurrence, and hospital stay were very close to the rates documented in open emergency repair.

To clarify this debate, Sartori et al. in a recent systematic review [88], analyzed the outcomes of the laparoscopic and open approach for the treatment of acute incarcerated groin hernia. The laparoscopic approach showed better results than the open repair in terms of hospital stay (4.8 ± 2.2 and 11 ± 3.1 days, *p* = 0.008), postoperative complications (9.8% vs 24.3%, *p* = 0.06), conversion rates (1.2% vs. 8.1%, *p* = 0.0023). It should be noted that laparoscopic repairs were converted to open repair (inguinotomy), whereas open repairs required conversion to laparotomy. Furthermore, at mean follow-up of 21.2 ± 6.7 and 17.2 ± 6.8 months (*p* =  < 0.0001), respectively, no statistically significant differences in recurrences rate were observed after laparoscopic and open repair (1.2% vs. 1.3%, *p* = 0.96). Long-term effectiveness of the TAPP approach in emergency setting was also supported by Zanoni et al. In this cohort study, no recurrence or severe complications were reported after 4 years of follow-up [89].

#### Ventral and incisional Hernias

Open ventral hernia repair is associated with significant morbidity, especially for large or complicated hernias requiring extensive dissection. As shown for inguinal hernias; similarly for ventral and incisional hernias, the laparoscopic repair is associated with faster recovery time, less wound infection and lower rates of chronic pain, without compromising the repair durability [90–93]. 

Shah et al. in 2008 performed a retrospective study that demonstrated the safety, feasibility and low complication rates of laparoscopic ventral abdominal wall hernia repair, even for incarcerated hernias [94], concluding that careful bowel reduction with adhesiolysis and mesh repair, in an uncontaminated abdomen, with a 5-cm mesh overlap, were key factors for a successful outcome. Two large retrospective studies for hernia repair in emergency settings, had also demonstrated shorter lengths of stay and fewer infections and decreased wound morbidity following laparoscopic ventral hernia repair in comparison to the open approach [95, 96].

#### Combined ventral-incisional and groin hernia

Jacob et al. compared short and long-term outcomes of laparoscopic and open approaches on a cohort of adult patients who underwent emergency surgery for acutely incarcerated/ strangulated ventral and inguinal hernias [97]. This study had a mean follow-up of 2 years. No difference in recurrence rates were reported. A total of 13% of the laparoscopic patients required visceral resection, yet none of these patients developed mesh infection. Operative times and lengths of stay were significantly shorter in the laparoscopic group, and long-term results showed better outcomes in terms of rest pain, difficulty during exercise and local discomfort. When investigating the reasons driving surgeons to choose the open approach, most mentioned personal preferences and lack of sufficient laparoscopic skills are the main causes. A total of 22% of surgeons mentioned hernia size as the deciding factor (in spite of hernia defects smaller than 7 cm in these cases), and only 23% stated objective factors such as patient instability or hernia size larger than 10 cm as the cause to choose an open approach.

Not all cases are amenable to laparoscopic repair, and cases involving unstable patients or very large hernia defects were still best suited for the traditional open approach. However, most cases can be completed laparoscopically with better results in the short and long-term. The laparoscopic approach also allows the surgeon to perform bowel resection if the segment is deemed non-viable after the repair has been completed, with adequate time given to the bowel to manifest viability after reduction. Moreover, in case of bowel resection, the mesh can still be placed in a different compartment, thus minimizing the risk of contamination**.**

The Accreditation and Certification of Hernia Centers and Surgeons (ACCESS) Group of the European Hernia Society (EHS), recognizes that there is a growing need to train specialized abdominal wall surgeons through clinical fellowships, in view of the increasing complexity of abdominal wall surgery and new MIS techniques [98].

### Laparoscopic approach in Adhesive Small Bowel Obstruction (ASBO)

Post-operative adhesions are the leading cause of small bowel obstruction, accounting for 60% of cases [99]. Complications of postoperative adhesion formation are frequent; thus, adhesive small bowel obstruction (ASBO) is a significant contributor to short and long-term postoperative morbidity and mortality with significant negative effects on patients’ health and functional decline [16, 100, 101], especially in elderly patients [102]. ASBO and its treatment greatly increases the workload in clinical practice, with a strong impact on in-hospital and associated health care costs [103–105]. Although 75% of cases of adhesive SBO will initially undergo non-operative management, up to half of these patients will fail this approach and require surgery [106]. Morbidity, length of stay (LOS) and all the aforementioned consequences are mainly affected by the need for surgical intervention. Average hospitalization was tripled (16 vs. 5 days) when surgery was required and associated estimated costs were seven times higher (€16 305 vs. €2 227) in a Dutch study in 2016 [107]. Operative management of a first episode of ASBO might reduce the risk of readmission compared with non-operative management (13% vs. 21% after a median of 3.6 years follow-up) [108]. However, recurrence rates for an episode of ASBO remain high [109].

Historically, abdominal exploration through laparotomy has been the standard surgical approach to ASBO. Laparoscopy has not only been shown to have a protective effect in the development of ASBO [110–113], but several studies in the last decades support laparoscopic adhesiolysis as a new surgical approach to ASBO, with potential benefits such as faster recovery, less pain, and fewer recurrences [114–117]. The implementation of laparoscopic surgery for ASBO however, is slow. Although the laparoscopic approach is currently used more frequently [118], only 50–60% of surgeons would consider using it for small bowel obstruction according to surveys from the UK and the USA [119, 120].

Lack of widespread adoption could be due to three major reasons: laparoscopic adhesiolysis is technically demanding [121], it has been associated with higher risk of iatrogenic bowel injury [122] and controversies existed about its role in reducing the risk for future recurrences [123].

Several earlier systematic reviews and meta-analyses of non-randomized studies [124–126], have found a substantial decrease in morbidity, mortality, wound infections, and LOS in patients who had laparoscopy compared with open surgery. These findings are significant despite these earlier studies were influenced by selection bias. The 2017 update of the evidence-based guidelines from the WSES ASBO working group recognized the role of laparoscopic adhesiolysis in reducing morbidity, in selected cases of ASBO requiring surgery [109].

An international, multicenter, randomized, open-label trial (LASSO trial) compared laparoscopic versus open adhesiolysis for ASBO not resolved with conservative management [127]. Laparoscopic adhesiolysis provided quicker recovery and return of bowel function, with reduced use of epidural catheters than open surgery and no differences in morbidity, mortality, wound infections and rates of bowel injuries were found between the two groups. The trial reported only short-term results, but the laparoscopic approach might have additional benefits in the long term. Patients with anesthetic contraindications, such as hemodynamic instability were excluded from the trial, and inclusion criteria enabled selection of patients who had a high likelihood of having a single adhesive band causing the obstruction (no confirmed or suspected peritoneal carcinosis, known wide adhesions, previous open surgery for endometriosis/generalised peritonitis, abdominal malignancy, previous radiotherapy of the abdominal region, three or more earlier open abdominal operations, suspicion of other source of obstruction than adhesions, recent abdominal operation, previous laparotomy for aorta or iliac vessels, or Crohn’s disease).

Results from a recent systematic review and meta-analysis [128] showed that laparoscopic adhesiolysis for ASBO was associated with a decrease in 30-day mortality, LOS, operative time, time to flatus, risk of severe postoperative complications, and early unplanned reoperations, when compared to open approach, with no difference in iatrogenic bowel injury. Given the methodological limitations of the unmatched studies, these results might be attributable to patient selection, but improving patient selection may broaden the population that can benefit from the known benefits of laparoscopy.

In the absence of hemodynamic instability or contraindications for pneumoperitoneum such as cardiopulmonary failure or severe bowel distention or severe intra-abdominal sepsis due to peritonitis [129–131], patients could potentially benefit from a laparoscopic approach. There is consensus that potentially one of the eligibility criteria for the laparoscopic approach would be the presence of either a single band or a limited extent of adhesions [127, 131]. However, a previous midline laparotomy, the suspicion of bowel strangulation/ volvulus or bowel ischemia and CT findings of moderate small bowel distension, should not be seen as strict contraindications for a laparoscopic approach [131]. Some of these factors could be controlled by matching in nonrandomized studies. It is important to note that it can be difficult to control or report on the laparoscopic skills of the surgeon and experience of the full operative team as this can intuitively affect outcomes. Efforts should be made to increase the laparoscopic technical experience of emergency surgeons and to address a tailored surgical management of ASBO. In a stable patient, a careful step-by-step laparoscopic technique is safe overall and should be recommended.

### Laparoscopic approach in Colo-rectal emergencies

Over one-third of acute surgical admissions are for colorectal pathologies, including diverticular, malignant and inflammatory bowel diseases [132, 133].

A systematic review of twenty-two comparative studies and case-series, compared outcomes of laparoscopic versus open colorectal emergency resections [134]. Except for an expected longer operating time, the laparoscopic approach was associated with a significantly lower complication rate and LOS. Despite the benefits in terms of lower mortality, morbidity, LOS and hospital costs, a large population-based study in the USA published two years later, found that less than 5% of urgent and emergent colectomies were performed laparoscopically [135].

#### Colo-rectal cancer emergencies

In the 2017 WSES guidelines on colon and rectal cancer emergencies stated that the use of laparoscopy in the emergency treatment of obstructed left-side colon cancer (OLCC), cannot be recommended and should be reserved for selected favorable cases and performed preferentially in specialized centers (LoE 4-GoR C) [136].

It is known that the use of stents as a bridge to surgery could increase the odds of laparoscopic resection, allowing better short-term outcomes than upfront emergency surgery, with significantly lower stoma rates [137].

Due to controversies about long-term outcomes, the 2017 WSES guidelines stated that stents could not be considered as the treatment of choice in the management of OLCC but may represent a valid option as a bridge to surgery in selected cases and in tertiary referral hospitals [136]. A recent meta-analysis [138] found that colonic stenting and decompressing stoma strategies as a bridge to surgery is associated with better 5-year overall survival and disease-free survival rates than upfront emergency resection. Data related to patients’ clinical status were not taken into account, but these findings support the recommendation that stable patients with obstructed colon cancer may benefit from a laparoscopic approach and a decompressive stoma or colonic stenting, allowing for higher rates of subsequent minimally invasive resection.

Regarding obstructed right-sided colon cancer, the 2017 WSES guidelines considered an upfront right hemicolectomy with primary anastomosis as the preferred option. A terminal ileostomy associated with a colonic fistula represents a valid alternative if a primary anastomosis is considered unsafe (LoE 2-GOR B) [136].

Nevertheless, a recent meta-analysis has shown that preoperative colonic stenting for right-sided malignant large bowel obstruction, can be considered in select cases [139]. This may further increase the rates of laparoscopic resection in the emergency settings for right-sided obstructive colon cancer.

#### Complicated acute diverticulitis

The 2020 update of the WSES guidelines for the management of acute colonic diverticulitis in the emergency setting, advises for an emergency laparoscopic sigmoidectomy, if technical skills and equipment are available, in patients with diffuse peritonitis due to perforated diverticulitis (weak recommendation based on low-quality evidence, 2C) [140]. In selected unstable patients, damage control surgery (DCS) with staged laparotomies, is instead suggested to facilitate both the severe sepsis control as well as potentially improving the rate of primary anastomosis [141]. A recent analysis in the USA showed that laparoscopic sigmoid resection was associated with lower morbidity, shortened LOS and fewer complications when compared to open surgery. Nevertheless, a low rate of laparoscopic-first approach (11.4%) and a high conversion rate (38.6%), were reported. Conversion, although frequent, didn’t increase mortality and morbidity when compared to an upfront open approach [142]. This corroborates results previously obtained in a similar study by Lee et al. [143], confirming that training efforts to increase the adoption of MIS and to decrease conversion rates, are justified. Laparoscopy was recommended as the appropriate surgical approach in hemodynamically stable or stabilized patients with diffuse peritonitis due to perforated diverticulitis by Nascimbeni et al. This was a recent multidisciplinary review and position paper on the management of perforated diverticulitis with generalized peritonitis [144].

Zhang et al., [145] in a 2022 systematic review and meta-analysis, compared laparoscopic versus open Hartmann’s procedure in clinically suitable patients. The laparoscopic approach allowed for a shorter LOS, and a lower risk of overall surgical site infections. The single-arm analysis of the laparoscopic Hartmann procedure also showed an unprecedented high colostomy reversal rate by more than 80%.

It remains unclear what the real benefits of laparoscopic lavage are in Hinchey III Diverticulitis compared to sigmoid resection [146]. Long-term follow-up of a RCTs (Scandivian Diverticulitis) [147] and a systematic review of 3 RCTs [148] conducted in the last decade showed no differences in severe complications and mortality, despite recurrence of diverticulitis after laparoscopic lavage was more common and often leading to sigmoid resection (especially within 30 days postoperatively).

Another systematic review and meta-analysis [149] found no difference in terms of postoperative mortality and early reoperation rate but significantly higher rate of postoperative intra-abdominal abscess in patients who underwent laparoscopic lavage compared to those who underwent surgical resection.

The increased risk of reoperation or postoperative abscess formation must be weighed against the lower stoma incidence in the laparoscopic lavage group. Azhar et al. in a recent a post hoc analysis of the SCANDIV trial and LOLA arm of the LADIES trial [150], found that active smoking status and corticosteroid use were risk factors for laparoscopic lavage treatment failure. Shared decision-making considering patient risk-factors and both short-term and long-term consequences is encouraged, to individualize treatment in such cases.

#### Re-laparoscopy for secondary emergencies

Re-laparoscopy for managing complications following laparoscopic colorectal surgery appeared to be safe and effective in selected stable patients, as shown by Chang et al. [151] in a systematic review and meta-analysis of 11 studies. The commonest indication for re-laparoscopy was anastomotic leakage (74.3%), followed by postoperative hernia and adhesions with small bowel obstruction and colonic ischemia. Four out of the 11 studies compared the outcomes of re-laparoscopy versus open re-interventions and found that the laparoscopic approach was associated with reduced intensive care unit length of stay (ICU-LOS) and overall reduced hospital stay, quicker resumption of normal diet and time to normal stoma activity, as well as reduced morbidity and mortality.

The benefits of the laparoscopic approach were confirmed by Fransvea et al. [152] in a recent systematic review and meta-analysis of 19 studies involving 1394 patients who required reoperation for the treatment of complications following laparoscopic primary colorectal surgery. In 38.2% of these patients, a laparoscopic approach was adopted. The most common indication was anastomotic leakage and the most common type of intervention performed in the re-laparoscopy group was diverting stoma with or without anastomotic repair/redo (47.1%). A significantly shorter mean LOS with a lower risk of mortality was observed in the re-laparoscopy group than in the redo-open group.

Some studies subsequently supported re-laparoscopy in expert hands and in selected hemodynamically stable patients [153, 154]. However, these results may be realized only with adequate laparoscopic expertise [155], and a recent study found that a total of 50 laparoscopic reoperations might be needed to achieve an appropriate learning curve with reduced operative time and lower conversion rates [156].

#### Iatrogenic colonoscopy perforation (ICP)

An increasing number of screening, diagnostic, and therapeutic colonoscopies are being performed every year with therapeutic colonoscopies generally associated with a higher risk for ICP considering the age and the increase of comorbidities in patients that undergo colonoscopy, and current endoscopic excision/resection techniques with deeper wall dissection [157]. Emergency surgery is indicated as the first line treatment in patients with ongoing sepsis, signs of diffuse peritonitis, large perforations, and failure of conservative management and in the presence of certain concomitant pathologies, such as unresected polyps with high suspicion of being a carcinoma [158].

The 2017 WSES guidelines for the management of iatrogenic colonoscopy perforation recommend laparoscopy as the preferred first-line surgical approach for the management of ICP. Conversion should be considered whenever necessary regarding the ability of the operator to proceed laparoscopically, the tissues viability, and the patient’s overall status. Patient instability was considered as the only absolute contraindication to a laparoscopic-first approach [159].

### Laparoscopic approach in acute mesenteric ischemia (AMI)

Traditionally, AMI has been treated with open surgery. Over the past two decades, the development of endovascular techniques has made this approach an alternative for patients with occlusion of the superior mesenteric artery (SMA). There has been an absence of randomized controlled trials due to the strong heterogeneity and physiological diversity of patients with AMI [160], and controversies regarding the use of endovascular techniques as the primary management of AMI [161]. Nonetheless, in a systematic review and meta-analysis [162] El Farargy et al. have shown that endovascular therapy is associated with lower rates of mortality and bowel resection than the traditional, open approach.

AMI course has three stages: ischemia, necrosis, peritonitis. There is a discrepancy between subjective pain and objective tenderness and intraoperative findings: this relates to the timeline of the ischemic process. In the initial stages of ischemia surgical inspection of the bowel itself is not informative, as the ischemia starts from the mucosa toward the serosa and bowel is peristaltic, not black, the peritoneum is shiny which can lead to a false negative result.

In hemodynamically stable patients, without CT findings of transmural necrosis/overt peritonitis, endovascular revascularization procedures are the primary option in cases of arterial occlusion if the necessary expertise is available as recommended by the 2022 WSES guidelines update on Acute Mesenteric Ischemia [163].

After a period of ischemia of greater than 2 h, a transmural intestinal infarction develops [164]. If the physical examination demonstrates signs of peritonitis, there is likely irreversible intestinal ischemia with bowel necrosis and prompt laparotomy was recommended by the previous 2017 WSES guidelines [165]. The goal of surgical intervention for AMI includes: re-establishment of the blood supply to the ischemic bowel, resection of all non-viable regions, preserving of all viable bowel.

The 2012 European Association for Endoscopic Surgery (EAES) consensus for the laparoscopic approach to the acute abdomen stated that there were no published data demonstrating significant advantages of laparoscopy in the diagnosis and treatment of acute bowel ischemia [34].

Patients with peritonitis on the basis of AMI are frequently unstable, however the 2022 WSES guidelines update has proposed laparoscopy as alternative to laparotomy in hemodynamically stable patients [163]. Laparoscopy has the ability to verify intestinal viability promptly and accurately and it is crucial in patients with AMI because being the most important factor influencing outcome. Non-viable bowel, if unrecognized, results in multisystem organ dysfunction and death. However, the utility of the laparoscopic approach has recently been confirmed not only to verify the diagnosis in dubious cases, but also to evaluate the extent of the ischemic small bowel segment and to offer a treatment option in cases of segmental necrosis due to embolism. Moreover, when a second-look surgery is indicated, second-look laparoscopy may be a useful alternative to conventional surgery with the advantages to avoid the trauma and risks of re-laparotomy in critically ill patients and the opportunity to be performed as an ICU bedside procedure [166]. Several studies supported the laparoscopic approach for the evaluation of Non-Occlusive Mesenteric Ischemia (NOMI) in post-cardiac-surgery patients [167, 168] and its utility in distinguishing ischemia from reperfusion injury [169], where CT-scan can be frequently equivocal.

In addition to traditional surgical inspection of the bowel, some techniques have been proposed to aid intraoperative assessment of bowel viability. Some of these techniques rely on bowel oxygenation, myoelectric activity and bowel perfusion. Flowmetry with fluorescein dye is currently considered the first-line adjunctive tool for intraoperative assessment of bowel perfusion, using a Woods Lamp in open laparotomies or laparoscopically using an endoscope with appropriate filters [170, 171].

Indocyanine Green (ICG) utilization in the emergency setting, particularly in AMI, has not been well investigated to date. Early animal models and isolated cases have shown promise, being able to detect ischemia that was not diagnosed on pre-op CT. ICG might also be useful in predicting delayed intestinal ischemic complications and can assist in deciding the resection margins [172–175]. Sequential case series have found that emergency intraoperative bowel viability assessment with ICG, helps to preserve bowel length and to define resection margins better than clinical judgment alone in 35% of cases [176, 177]. Comparative studies, and ultimately a prospective trial, are warranted to re-evaluate intraoperative assessment techniques for bowel viability in AMI, based on metrics that account for efficacy and clinical utility. These recent positive results also suggest the importance of revisiting the current standard of care, which holds fluorescein flowmetry as the first-line adjunctive tool for surgical decision-making [178]. Theoretically, as a near-infrared fluorophore, ICG should outperform fluorescein when assessing bowel viability due to decreased background and greater tissue depth of penetration, and it is currently more available and easily integrated into existing laparoscopic equipment.

### Laparoscopic approach in perforated peptic ulcer (PPU)

A laparoscopy-first approach in stable patients with perforated peptic ulcer (PPU) was suggested by the 2020 WSES guidelines on perforated and bleeding peptic ulcers. An open approach was recommended in the absence of appropriate laparoscopic skills and equipment and in hemodynamically unstable patients [179].

A meta-analysis by Cirocchi et al. [180] compared laparoscopic to open surgery for patients with PPU, including eight RCTs for a total of 615 patients (307 laparoscopic and 308 open repair). Although the included studies had a comprehensible risk of bias, the comparison reported a significant advantage of laparoscopic repair with less postoperative pain in the first 24 h after surgery and less postoperative wound infections. No significant differences between laparoscopic and open surgery were found in overall postoperative mortality, the suture leaks, intra-abdominal abscesses and reoperation rates.

Traditional surgical management of simple PPUs involved laparotomy with primary suture closure or omental patch [181]. The first laparoscopic treatment of a perforated peptic ulcer with an omental patch and fibrin glue was described in 1990 by Mouret et al. [182]. A recent systematic review and meta-analysis comparing postoperative outcomes of laparoscopic versus open omental patch repair of PPU [183] included a total of 29 studies with 5311 patients and four RCTs with 238 patients. Most of the ulcers were in the duodenum (57.0%) followed by the stomach (30.7%). Mean ulcer size ranged from 5 to 16.2 mm in laparoscopic repair and 4.7 to 15.8 mm in open repair. Laparoscopic repair was associated with lower 30-day mortality, overall morbidity, surgical site infection, and LOS.

There is increasing adoption of the laparoscopic approach with decreasing conversion rates, as shown by Coe et al. [184] in a recent analysis on a total of 5253 patients who underwent PPU repair from December 2013 to December 2017, using data from the National Emergency Laparotomy Audit. Across the 4-year study period, laparoscopic repair increased from 20 to 26% and the conversion rate decreased from 40 to 31%. A recent retrospective study compared the outcomes of patients who received different surgical approaches for PPU [185]. In the open approach group, in-hospital mortality and need for post-operative ICU were significantly higher, and the postoperative stay was longer. Previous abdominal surgery, ulcer size, and a posterior ulcer location, were predictive factors for conversion to an open approach.

A tailored approach is suggested by the 2020 WSES guidelines [179]. The surgeon must consider the ulcer location and the size (considering open repair for ulcers larger than 2 cm). For large gastric ulcers that raise the suspicion of malignancy, resection with operative frozen pathologic examination is suggested. In cases of large duodenal ulcers, the surgeon has also to consider the need for resections or repair plus/minus pyloric exclusion/external bile drainage.

Shelat et.al proposed some selection criteria for surgical training in laparoscopic PPU repair (Boey score of 0 or 1; ulcer size less than 10 mm; located in pyloro-duodenal area; no suspicion of malignancy; no previous abdominal surgery; ASA < 3) as being effective to reduce aspects of the learning curve and to enhance patient safety [186].

#### Video-assisted retroperitoneal debridement in severe acute pancreatitis (AP)

Approximately 10–20% of patients with AP develop pancreatic necrosis, and about one-third of them will develop infection of the necrotic tissue [187]. While sterile necrosis is associated with a 5–10% mortality, the mortality rate increases to 20%-30% when infection occurs. Patients with infected pancreatic necrosis may require radiologic, endoscopic or surgical intervention in up to 40% of cases [188]. The 2019 WSES guidelines on severe acute pancreatitis recommended percutaneous drainage as the first line treatment (step-up approach) in infected pancreatic necrosis. It defers the surgical treatment to a more favorable time or even results in complete resolution of the infection in 25–60% of patients. MIS strategies, such as transgastric endoscopic necrosectomy or video- assisted retroperitoneal debridement (VARD), resulted in decreased postoperative new-onset organ failure, despite the need for more interventions. Moreover, in stable patients with severe acute pancreatitis, an open abdomen has to be avoided if other strategies can be used to mitigate or treat severe intra-abdominal hypertension [189].

An international audit, the MANCTRA-1 (coMpliAnce with evideNce-based cliniCal guidelines in the managemenT of acute biliaRy pancreAtitis) [190], has showed an overall poor compliance with evidence-based management guidelines, with wide variability depending on the admitting specialty. It analyzed data from 5275 consecutive patients admitted to any of the 150 participating general surgery, hepato-pancreatobiliary surgery, internal medicine, and gastroenterology departments with a diagnosis of acute biliary pancreatitis between the beginning of 2019 and the end of 2020. Only 33.7% of patients with infected pancreatic necrosis underwent a step-up approach as their first treatment, rather than upfront surgery and only 37.2% of them underwent treatment after four weeks of symptom onset, as recommended by guidelines. Although the adherence to guidelines for acute pancreatitis were low, compliance can reduce mortality, shorten hospital stay and reduce costs [191].

Recently Podda et al. developed a new care bundle for managing patients with acute biliary pancreatitis using an evidence-based, artificial intelligence (AI)-assisted GRADE method (The 2023 MANCTRA Acute Biliary Pancreatitis Care Bundle) [192]. In regard to clinically deteriorating patients with acute necrotizing pancreatitis, associated with or without infected necrosis, the evidence consistently supports the use of the endoscopic step-up approach as the first interventional therapeutic approach. The minimally invasive surgical step-up approach is considered as the alternative choice.

### Laparoscopic approach in abdominal Trauma

Trauma is the main cause of death during the first half of the human lifespan and the fifth leading cause of death in all age groups, resulting in a major impact on global public health [193]. Laparotomy has traditionally been considered the standard surgical approach for abdominal trauma but it is associated with morbidity ranging from 20 to 41% [194–196]. Advancements of imaging technology and selective nonoperative management strategies have led to a decrease in non-therapeutic laparotomy for hemodynamically stable abdominal trauma patients [197–201]. They should first undergo a contrast enhanced CT scan [202] and non-operative management (NOM) can be the initial approach in most cases, especially in blunt trauma and in the management of solid organ injury [203, 204], without hollow viscus and mesentery injuries or signs of viscus perforation.

Nevertheless, about 25% of all abdominal trauma cases will require surgical abdominal exploration or treatment [205, 206] for NOM failure or missed abdominal injuries, that remains significantly present and cause of important morbidity and mortality. On the other hand, laparotomy related morbidity, especially in negative cases, remains significantly present and is associated with high complications rates and prolonged hospital LOS [207], further increased in case of temporary abdominal closure for second-look purposes [208].

Laparoscopy was first used as a diagnostic tool, especially to exclude peritoneal violation or occult diaphragmatic injury in hemodynamically stable patients with penetrating abdominal trauma, mostly in case of anterior/flank stab wounds or tangential gunshot wounds [209, 210]. In this setting, diagnostic laparoscopy has been shown to be effective and to reduce the negative laparotomy rate [211–214]. In penetrating wounds to the upper abdomen and lower precordium, laparoscopic transdiaphragmatic pericardial window was found to be a safe and effective modality to evaluate hemodynamically stable patients who are at risk for both cardiac and abdominal injuries [215, 216]. However, a diagnostic laparoscopy in penetrating abdominal trauma stable patients could not be recommended “a priori” [217], but should be evaluated on CT scan findings and clinical situation.

It must be noted that in the past, a higher rate of missed injuries was reported by some studies [218, 219]. In a 2013 meta-analysis by O’ Malley et al., a total of 2569 patients underwent diagnostic laparoscopy. A total of 1497 out of 2569 avoided a non-therapeutic laparotomy thanks to the use of diagnostic laparoscopy. Although, eventually 83 missed injuries were reported, mainly by a subsequent laparotomy performed in case of identification of peritoneal violation or lesions that required surgical repair [219]. However, most of studies were retrospective, and many were simple audits of the authors’ early experience with laparoscopy. In addition, there is a lack of standardization of laparoscopic technique. In some studies laparoscopy was solely used as a screening tool prior to laparotomy, without completing a full laparoscopic examination. Recent literature however showed that with the selected use of preoperative imaging, advances in technology, improvement in surgical technique and experience, along with the development of a systematic standardized laparoscopic examination protocol with adherence to the predetermined steps of a procedure, missed injury rates dropped and therapeutic procedures rate increased [220–222]. In a 2015 systematic review and meta-analysis comparing laparoscopy to laparotomy for the management of penetrating abdominal trauma on a total 3362 patients [223], Hajibandeh et al. found that laparoscopy was associated with a significantly lower risk of wound infection and pneumonia and a significantly shorter LOS and procedure time with no difference in missed injuries compared with laparotomy. Only one of the included studies was a RCT, and these results came mainly from retrospective and prospective cohort studies. However, the meta-analysis demonstrated that the laparoscopic evaluation of hemodynamically stable patients was safe and reduced post-operative complications.

The utility of laparoscopy in patients sustaining blunt abdominal trauma has conversely received only minor attention [224], and its therapeutic role is unclear due to a paucity of studies and learned opinion. However, some studies have shown that since its application in cases of blunt trauma, the rate of negative laparotomy has further decreased, and the laparoscopic approach has proven to be safe and effective for hemodynamically stable patients with blunt hollow viscus and mesenteric injuries, when conducted by experienced surgeons [225, 226]. In a systemic review and meta-analysis of 19 studies including a total of 1520 hemodynamically stable blunt abdominal trauma patients requiring surgery [227], Ki et al. aimed to evaluate the usefulness of therapeutic laparoscopy. The laparoscopic approach showed favorable outcomes in terms of blood loss during surgery, hospital stay, missed injury, decreasing nontherapeutic laparotomy rates and morbidity. The conversion rate has decreased in the most recent studies.

There is increasing evidence supporting the laparoscopic approach can be performed safely whether injuries are blunt or penetrating, given hemodynamic stability and proper technique. Patients may thus benefit from the shorter hospital stays, less pain, quicker recoveries, and low morbidity and mortality rates that the minimally invasive techniques afford [228–232]. More recently, some systematic reviews and meta- analysis [233, 234] have demonstrated the safety and effectiveness of the laparoscopic approach in hemodynamically stable patients considering both penetrating and blunt abdominal trauma, overall demonstrating the role of laparoscopy in avoiding non therapeutic laparotomy and its advantages in reducing postoperative complications and decreasing LOS. In a 2022 meta-analysis that compared laparoscopy to laparotomy outcomes, in a total of 5517 hemodynamically stable patients with penetrating and blunt abdominal trauma [234], Wang et al. found no differences in the incidence of missed injury and mortality rates in the two groups with similar risk of intra-abdominal abscesses, thromboembolism, and ileus. However, in the laparoscopic group there was a decreased incidence of wound infection and pneumonia with shorter hospitalization and procedure times.

In recent years, therapeutic laparoscopy has been increasingly adopted in patients with trauma. In a systematic review and meta-analysis including 9817 laparoscopies performed for abdominal trauma; only 26.2% of the cases were converted to a laparotomy. The incidence of therapeutic laparotomies showed a reduction from 69 to 47.5%, whereas the incidence of therapeutic laparoscopies increased from 7.2 to 22.7% [235]. This evolution was possible because of considerable improvements made in laparoscopic skills and equipment over the past few decades. The 2022 WSES guidelines on blunt or penetrating bowel injury [236] stated that in hemodynamically stable patients, laparoscopy can be used, and the bowel injuries identified can be treated laparoscopically, based on the surgeon’s experience and logistics of the trauma center. Other recent studies demonstrated the feasibility of laparoscopic procedures such as bowel resection, bowel repair, bladder repair, splenectomy, distal pancreatectomy, diaphragm repair and hemostasis, thus addressing a wide range of indications for the adoption of a laparoscopic approach in stable patients [237–242]. Hemoperitoneum, peritoneal penetration or retroperitoneal organ injury were associated with a significant risk of conversion [242, 244]. Thus, trauma surgeons need experience and advanced skills to appropriately use this technique, so efforts should be made for the implementation of laparoscopic training for trauma surgeons.

Laparoscopy reduces hospital costs in abdominal trauma patients [245], but lack of resources (operating room availability and trained personnel) are barriers to the adoption of emergency laparoscopy in low-resource settings [246]. Gomez et al. investigate the feasibility and safety of therapeutic laparoscopy in the management of stable penetrating abdominal trauma patients requiring surgery in Colombia, comparing the minimally invasive approach to a laparotomy-first approach [247]. There were no missed enterotomies in the laparoscopy group. Surgical time and bleeding were significantly lower in the laparoscopic approach group and the time to oral intake and ICU-LOS was significantly shorter in the laparoscopic group. Although the study sample was small, these data suggest that emergency laparoscopic surgery may be safe, feasible, and effective in low-middle income countries, although it often remains limited in its accessibility, acceptability, and quality. Surgeons, policymakers, and manufacturers should focus on plans for sustainability, training and retention of providers, to further develop laparoscopy in this field [248, 249].

### Laparoscopy limitations and barriers

The effects of increased intra-abdominal pressure and hypercarbia due to carbon dioxide insufflation during laparoscopy, are well documented The necessary use of pneumoperitoneum and extreme patient positioning (e.g. Trendelenberg) may result in metabolic, respiratory, cardiovascular and neurological changes which might be deleterious in hemodynamically unstable patients requiring surgery, who are currently precluded from a laparoscopic approach [250–254].

Heart disease is not an absolute contraindication for laparoscopic surgery. However, low-pressure insufflation should be maintained in the case of cardiac dysfunction [34, 255]. The most pronounced cardiovascular effects can be seen when pneumoperitoneum is induced, with hemodynamic effects that are irrelevant in ASA I–II patients with 12–14 mmHg of carboperitoneum, while they are relevant in ASA III–IV patients, who need at least arterial pressure invasive measurement [256].

In patients with pre-existing pulmonary disease or severe obesity (BMI > 40), protective ventilation can correct hypercapnia, increasing minute volume, but avoiding excessively high insufflation volumes and pressures that may cause lung damage or may increase the risk of developing pulmonary complications. The patients suffering from pre-existing cardiopulmonary disease or chest trauma therefore need more intraoperative intervention to optimize mechanical ventilation and require invasive hemodynamic monitoring, using at least a radial arterial catheter to hematic gases and blood pressure continuous monitoring [257–259].

When hemodynamical change or ventilation problems arise, the surgeon and anesthetist, as a team must assess and correct the surgical laparoscopic settings if possible or to decide whether to continue a surgical procedure or to discontinue it and covert. Devices for intermittent pneumatic compression of the lower limbs can be used to reduced venous stasis, the reverse Trendelenburg position plays a part in reducing operation time, and, finally, stabilizing intravascular volume is beneficial.

Pneumoperitoneum and laparoscopy have been shown to result in a rise in intracranial pressure (ICP). Consequentially, laparoscopy should be used cautiously in patients who present with baseline elevated ICP or head trauma. In these patients, IAP must be kept as low as possible and ICP monitoring should be evaluated [260–262].

Gas embolism is a rare but dangerous complication that has been reported during laparoscopy by direct insufflation of CO2 into a vein or in an abdominal organ due to accidental insertion of the Veress needle or trocar [263]. The overall incidence of gas embolism during laparoscopic surgery is considerably low, at approximately 0.15% [264]. It is arguably even more likely in trauma patients with intra-abdominal venous injuries, especially in liver lacerations and presence of hypovolemia. The absorption of carbon dioxide (which may cause metabolic and hemodynamic changes such as acidosis, cardiac suppression, atelectasis, and increased intracranial pressure) may rarely lead to life-threatening consequences. The management includes the interruption of CO_2_ insufflation and the adoption of Trendelenburg position lying on the left side, starting hyperventilation with 100% FiO_2_. A central venous catheter or a catheter in the pulmonary artery to aspirate the gas and external cardiac massage may be helpful. In the case of a massive embolism, cardiopulmonary bypass may be performed. Hyperbaric therapy should be considered. Once the patient has been stabilized, pneumoperitoneum can carefully be restored, but if the signs of cardiopulmonary imbalance remain, it may be necessary to convert to an open procedure [265–267].

Decompressive laparotomy is mandatory in abdominal compartment syndrome, in which laparoscopy cannot have by definition a therapeutic role. The adoption of laparoscopy is also limited by the presence of injuries requiring packing or abdominal wall tissue loss in abdominal trauma or by the inability to distend further an abdomen already very distended [268].

Surgeons’ experience and confidence to perform laparoscopy in the emergency setting, particularly in abdominal trauma, remains a major factor for technical dissemination and standardization. The systematic review conducted by Cirocchi et al. (2017), showed that the skill of the surgeon, reported only in 25.7% of the studies, was heterogeneous, making it difficult to assess the role of the surgeons’ experience in managing these patients [235].

There is also significant heterogeneity amongst existing studies, such as characteristics of the studied groups, indications for laparoscopy, trauma mechanisms, anatomical location of the lesion, the setting where the procedure was performed, surgeon’s skill, and technique. These variables make it difficult to properly standardize or categorize studies for comparison and research purposes [269, 270]. Surgeons’ skill heterogeneity is largely due to the lack of structured training [271]. Indeed, there is a substantial difference between learning open surgery versus laparoscopic surgery. The old saying, “see one, do one, teach one,” is no longer applicable.

Emergency laparoscopy requires expert camera navigation techniques, in an environment where blood or contents of hollow organs can often obscure the view. It also requires critical skills such as the ability to mobilize intra-abdominal organs or to perform bowel loop inspection ("running loops") quickly and safely to allow a proper identification of the injuries and management of them. Moreover, in addition to the management of intraoperative complications, laparoscopic suturing is an essential technique required in many advanced laparoscopic procedures [272]. The training of laparoscopic skills, specifically for trauma, has not yet been described in the literature. A recent WSES position paper on Training Curriculum in minimally invasive emergency digestive surgery, was recently updated to lay the groundwork for developing standardized curricula and training programs in emergency MIS [10]. Balancing the safety of current patients with the need to promote procedural competency for future surgeons, a progressive and adequate training plan, based on simulation [273–277], supervised clinical practice (proctoring) [278, 279], and surgical fellowships are required [10, 98]. Advanced laparoscopic skills can be acquired during elective procedures and transferred to the emergency and trauma settings [280]. All of these competencies are encouraged to be taught with multiple sequential, structured, repetitive training modules and sub-modules, to allow a rapid gain in proficiency in laparoscopic skills [281, 282]. Surgical proficiency should be maintained with a minimum caseload [283], and results should be evaluated by adopting a credentialing system to ensure quality standards.

### Emergency robotic surgery

Although robotic surgery is currently mainly used in the elective setting, in recent years, its application has been increasingly reported in the emergency setting. In a 2021 WSES position paper on robotic surgery in emergency setting [284], De Angelis et al. showed some evidence of promising results and overall feasibility of robotic surgery, especially in emergency colorectal surgery, urgent cholecystectomy, gastrojejunal ulcer repair and emergency ventral hernia repair. Among the included studies, a series by Ceccarelli et al. [285], showed that postoperative outcomes were good for robotic emergency hiatal hernia repair, and the authors suggested that the potential advantages of robotics over a conventional laparoscopic approach were mainly related to the surgeon’s comfort and precision during the intervention. However, experts still advocate for strict patient selection when considering emergency general surgery procedures with robotics; considering it only for hemodynamically stable patients and if adequate expertise is available.

Despite the perception that laparoscopy and robotic techniques are very similar in approach, views and dissection [286], several studies suggested that previous laparoscopic experience has a limited impact on robotic proficiency [287, 288]. This finding, associated with the shortened learning curve for robotic surgery, should encourage the adoption of this technology to approach technically demanding cases [289, 290].

In a recent study by Rifai et al. [291], two equally qualified surgeons performed both laparoscopic and robotic-assisted appendectomies and cholecystectomies over 2 years. Surgical times, hospital stay, rates of conversion to open procedure, and readmission within 30 days were evaluated. Intraoperative times for robotic appendectomy and cholecystectomy were not longer than laparoscopic approach. Furthermore, the robotic approach shortened the time to discharge and the likelihood of conversion to an open procedure. Curfman et al. compared the outcomes of over 2500 emergency sigmoid resections for diverticulitis with robotic, laparoscopic and open approaches [292]. Emergency robotic sigmoidectomy showed many benefits compared to open approach, with a significant decrease in ICU admission rates, anastomotic leaks, and reduced LOS. When compared to the laparoscopic approach, robotic sigmoidectomy showed similar outcomes but it was associated with a statistically significant improvement in anastomotic leak rates, respectively 4.5%, and 0.8% Furthermore, there was a striking difference in conversion rates. Laparoscopic cases were converted in over 28.7% versus 7.9% of robotic cases.

The elimination of physiological tremors, motion scaling, and improved ergonomics compared to laparoscopy, may contribute to facilitating the performance of some difficult procedural steps and reduce the risk of conversion. Laparoscopy is burdened by the physical stress of the surgical team, whereas robotic surgery offers a less physically demanding approach [293, 294]. Moreover, in robotic surgery, in-person mentoring can be performed if a second robotic console is present in the hospital (such as telestration or tele-assisted surgery). Despite the current skepticism, it is unquestionable that robotic surgery can have a pivotal role in developing telemedicine and telesurgery [295, 296]. Furthermore, robotic undocking, when emergency conversion is required, could be fast if adequate training of all surgical team members is pursued [297].

Nonetheless, concerns for the adoption of robotics in emergency surgery still persist in relation to its increased costs. A 2022 WSES survey among international acute care and emergency surgeons [298], found that 63% of emergency surgeons still did not have robotic surgery platforms in their institution and for those who had it, it was mainly used for elective surgery. Despite the fact that 25% of emergency surgeons were trained in robotic surgery, only 10% were currently performing it.

However, in addition to the Da Vinci Robotic Surgical System (Intuitive), the surgical marketplace was recently enhanced with several different robotic platforms either approved for human use, such as the CMR Versius (Cambridge Medical Robotics, Cambridge, UK) and the Distalmotion Dexter (Distalmotion, Epalinges, Switzerland) or under approval, such as the Medtronic Hugo (Medtronic Inc., Minneapolis, USA). It is to be expected that as with the trend in laparoscopic advances, that of the robotic platform will have increasing implementation in the field of abdominal emergencies.

### Single Recommendation

This comprehensive literature review and summary of the current scientific evidence has focused on evaluating the applications of laparoscopy in general surgery emergencies and abdominal trauma.

The international WSES experts panel, after a critical revision and discussion of the manuscript, has reached a consensus on a single position statement.

*Single Position Statement: *Laparoscopy is suggested to be considered as the first approach for stable patients requiring emergency abdominal surgery for general surgery emergencies or abdominal trauma *(Grade of recommendation: Strong recommendation, based on moderate-quality evidence, 1B).*

## Discussion

The present review summarizes the current evidence on laparoscopy and associated minimally-invasive procedures in general surgical emergencies and abdominal trauma. This paper also presents a shared consensus regarding the recommendations and indications for laparoscopy application in patients undergoing emergency abdominal surgery.

Over the last two decades, the role of laparoscopy in the emergency setting has increased considerably, as a result of improved surgical expertise and continued technological advances. Laparoscopy is well established as the standard of care for the surgical treatment of acute appendicitis and acute cholecystitis in stable patients, and guidelines have long recommended its first-line role in the absence of septic shock or absolute anesthetic contraindications [31, 33, 35, 43, 51]. Even in the presence of diffuse peritonitis or peritoneal contamination in perforated appendicitis, the benefits of the laparoscopic approach have been documented [43–46]. Previous upper abdominal surgery is no longer a contraindication to safe laparoscopic cholecystectomy [62]. Difficult gallbladders can be managed effectively through various laparoscopic bailout-techniques, to decrease conversion rates and associated morbidity, as reported in various recent systematic reviews [71–75]. A useful adjunct would be the use of ICG, which has been increasingly integrated into laparoscopic equipment [79].

There is an increasing amount of evidence demonstrating the benefits of the laparoscopic approach in other general surgical emergencies and abdominal trauma. Most ventral, incisional and groin hernias, even if clinically strangulated, can be treated laparoscopically with good results as shown by some recent studies [88, 89, 95–97]. The laparoscopic approach agrees to adequately evaluate the intestinal viability after hernia content reduction/repair. Although the traditional open approach is still preferable for unstable patients or hernias with very large defects. Stable patients with ASBO that require surgery, in the absence of cardiopulmonary impairment, severe bowel distension or severe intra-abdominal sepsis due to peritonitis and limited extent of adhesion, could benefit from a laparoscopic approach [127–131]. A careful, safe, systematic and reproducible step-by-step technique based on intraoperative findings could expand the population that will benefit from the advantages offered by a MIS, and potentially reduce future ASBO recurrences.The laparoscopic approach is recommended in the management of hemodynamically stable patients with generalized peritonitis from perforated diverticulitis in a recent multidisciplinary review and position paper [144]. Laparoscopic peritoneal lavage or even the resection, may be accomplished laparoscopically in centers with adequate expertise, as suggested by the 2020 WSES guidelines [140]. Although conversion remains frequent, some studies have shown that it is not associated with increased mortality and morbidity, when compared to upfront open procedures [141, 142]. Moreover, the colostomy reversal rate following Hartmann’s procedure is higher after a laparoscopic approach [145]. Some studies support re-laparoscopy in the management of complications following colo-rectal laparoscopic surgery, when performed in experienced hands and in selected hemodynamically stable patients [153, 154]. Recent meta-analyses demonstrated improved postoperative outcomes and lower mortality in re-laparoscopy cases [145, 152]. The 2017 WSES guidelines recommended to reserve the laparoscopic approach for selected favorable cases of obstructed left colon cancer [136]. However, the adoption of a decompressive stoma or SEMS as bridge to definitive surgery, could increase the odds of laparoscopic resection and reduce stoma rates with better short and long-term outcomes respect to open resection [138]. In stable patients with perforated peptic ulcer, a laparoscopic approach is suggested by the 2020 WSES guidelines [179]. Cirocchi et al. comparing laparoscopic and open approaches in cases of PPU, demonstrated that there were significant advantages from the laparoscopic repair with less pain and wound infections without significant differences in other postoperative outcomes however [180]. In stable patients with suspected bowel ischemia or overt peritonitis a laparoscopic approach can be performed as an alternative to laparotomy [163]. The laparoscopic approach has gained an important role in the management of acute mesenteric ischemia for its ability to confirm the diagnosis in doubtful cases, avoiding needless laparotomy in frail ICU patients. Moreover, bowel resection can be performed in cases of segmental necrosis and when a second-look operation is indicated, laparoscopy may be a useful alternative second-look strategy to open surgery [163, 166–168]. In stable patients undergoing emergency surgery for abdominal trauma, the laparoscopic-first approach was safe and effective. Several systematic reviews and meta-analyses over the last ten years [223, 227, 233, 234], demonstrated its safety in hemodynamically stable patients for both penetrating and blunt abdominal trauma. The application of laparoscopy has been associated with less laparotomies, allowing a reduction of postoperative complications and length of stays and there is a growing body of studies expanding the therapeutic possibility of laparoscopy in trauma [236–243] .

Despite this exciting development, the selection of a physiologically stable patient remains of paramount importance for a safe adoption of laparoscopic approach. This is reported by several current guidelines and studies [31, 33, 35, 36, 51, 97, 127, 129–131, 144, 145, 151, 153, 154, 159, 163, 179, 189, 209–213, 222, 223, 226, 228–230, 232–242, 247]. Trauma or critically ill patients requiring emergency abdominal surgery may be considered eligible for a laparoscopic approach if sustained stability of hemodynamic and respiratory parameters is achieved after resuscitation [299, 300]. While in stable patients the safety and effectiveness of the laparoscopic approach is strong, only few studies are available in hemodynamically unstable abdominal trauma patients [301]. Other studies such as those targeting “gasless laparoscopy”, if proven effective, could potentially offer a MIS technique without the intra-abdominal hypertension needed for the conventional laparoscopy [302, 303]. It could expand the indication of the laparoscopic approach to more severe patients.

Comparing laparoscopic to open approach in the emergency setting, the clinical and hemodynamic patients' status is not systematically reported [88, 128, 138, 152, 180, 233] and this can be a bias for patient’s outcomes evaluations. Although a comprehensive preoperative assessment is not always possible in emergencies, careful patient selection and a defined multidisciplinary operative plan shared by an anesthetist and surgeon is advised and should be reported in future studies.

Actually, the vast majority of emergency major abdominal surgery is still performed via laparotomy. Analysis from the USA and UK have shown that only about one out of five patients requiring major emergency abdominal surgery underwent a laparoscopic approach  [2, 304], as also declared by surgeons in a recent WSES survey [3]. However, the 7th NELA report has shown that patients undergoing emergency laparoscopy, required a reduced ICU-LOS; they have half the length of inpatient stay and a 30-day mortality a third of that of patients undergoing emergency laparotomy. It has been questioned whether this is an effect of selection bias, with laparoscopy reserved for the fittest and lowest-risk patients. However, when laparoscopic cases were propensity-matched with open cases at a population level, the risk of mortality was reduced by half in patients managed with laparoscopy [305]. A laparoscopic approach is not applicable to all abdominal emergencies and unstable patients should be managed with prompt laparotomy. However, these findings suggest that preferring an open approach in stable patients, that might successfully undergo a minimally invasive procedure, can have negative implications in patients’ recovery and associated healthcare utilization.

Long-term outcomes are rarely collected in studies, especially in the emergency setting [127]. In this regard, the advantages of a laparoscopic-first approach may eventually be even greater, mainly through the avoidance of some long-term sequelae of open surgery. A Lower incidence of chronic pain following laparoscopic groin hernia repair was reported in a recent meta-analysis [84]. The long-term effectiveness of TAPP approach, as well as ventral hernia repair in emergency setting was recently investigated in term of recurrence, complications, and local discomfort, with very encouraging results [88, 89, 97]. Reduced rates of incisional hernia or adhesions were found in comparative studies of laparoscopy versus laparotomy [115, 306]. Further studies focusing on long-term outcomes and patient-reported outcomes in emergency settings are needed.

Unfortunately, surgical patient selection remains critical. In spite of the fact that the major limiting factor in the use of MIS in emergency surgery is the shock condition, the choice of surgical approach is mostly influenced by surgeon experience (especially prior laparoscopic experience in elective practice and subspecialty) and estimated prolonged surgical duration, beyond other patient characteristics (such as prior surgery, body mass index and comorbidity). Moreover, these factors, and their weight in influencing the surgical approach’s decision-making, are rarely reported in studies [3, 97, 119, 190, 235, 307, 308].

Adequate patient selection is often complicated by fears that laparoscopy might result in further patient issues, technical difficulties and additional operative time [121], or iatrogenic injuries [122]. The potential increase in operative times is sometimes cited as a negative factor of laparoscopy, but it seems that this does not affect the benefits of MIS. Harji et al. reported longer laparoscopic operative time in a meta-analysis that compared laparoscopic colorectal emergency resection to the open procedure. However, the laparoscopic group still had a significant reduction in complication rates and LOS [134]. Longer operative times can be anticipated in MIS approaches to major abdominal emergencies such as colorectal cancer emergencies, especially regarding stable patients, where oncological resection should be pursued [136].

However, as for laparoscopic appendectomy and cholecystectomy [43, 46, 53, 54], an increase in laparoscopic skills, would likely lead to a reduction of operative time. Zhang et al. in a recent meta-analysis found no difference in operative times comparing open to laparoscopic Hartmann’s procedure, which allowed reduced LOS and surgical infection, and a higher colostomy reversal rate [145]. A faster fascial closure may result in reduced operative time with laparoscopy, as found in the recent LASSO randomized trial of laparoscopic versus open surgery for adhesive bowel obstruction [127].

Approximately one out of two patients requiring emergency gastro-intestinal surgery underwent conversion, after a laparoscopic-first approach  [14, 304]. The underlying pathology and the required procedure were the strongest factors associated with conversion in a recent analysis of the NELA database, despite surgeon subspecialties being a crucial factor [274]. Moreover, reasons for conversion in each case were mostly unknown. Some cases may have been started laparoscopically to establish a visual diagnosis before proceeding to planned laparotomy, or converted due to iatrogenic injury rather than technical difficulties, or even due to equipment failure. Unfortunately, reasons for conversions and associated outcomes are frequently not reported in studies [114, 127, 233, 235, 244]. Future prospective work is required to quantify the surgeons’ judgment on the appropriateness of patients and pathology for attempted laparoscopy, and reasons for abandonment or conversion.

Patients could benefit from the MIS advantages, regardless of the eventual success or conversion of the laparoscopic approach. Matching at a cohort level, patients who underwent a laparoscopic approach and converted to open surgery, to those who went straight to laparotomy, Pucher et al. found that converted patients had lower in-hospital mortality, duration of hospital stays, and reduced blood loss [305]. Studies on emergent sigmoid resection in perforated diverticulitis have found that conversion, did not increase mortality and morbidity compared to planned open procedures [141, 142]. Benefits despite conversion may be due to the laparoscopic approach of full abdominal exploration and visual confirmation of the diagnosis with reduced tissue trauma, allowing smaller and more targeted open incisions, especially if part of the surgical procedure could be completed laparoscopically. These further advantages, regardless of conversion, emphasizes the importance of submitting stable patients with abdominal surgical emergencies to a first-line laparoscopic approach.

The experience and expertise of the surgical team directly affect procedures times, outcomes, effectiveness of the laparoscopic approach and conversion rates in abdominal emergencies [213, 221, 231, 309, 310]. An effective organization, communication and synergy between the entire operating room staff, as well as adequate experience and competence of the team are also essentials elements in an emergency setting [311–313]. The success rate of MIS implementation in abdominal emergencies is finally determined by a multifactorial combination of these elements, thus training, proctoring and continuous education should involve the operating surgeon and the entire surgical team. Several training and educational issues have been documented in the review. On the other hand, surgery residents are graduating with increasing laparoscopic experience [314, 315] and an increasing number of residents are going on to complete MIS fellowships [10, 98, 316]. This likely resulted in an overall larger proportion of surgeons who feel prepared to perform advanced laparoscopic procedures each year, as more surgeons with advanced laparoscopic and robotic skillsets matriculate. The process of acquiring laparoscopic and surgical skills depends on many factors and appropriate training may be difficult for the lack of dry and wet lab facilities [317]. However, trainees with no accessibility to this resource may practice with “homemade” dry boxes, which are equally effective means of teaching laparoscopic skills to novice learners, when compared to simulators [275]. Internet-based technologies have dramatically changed the interaction between trainees and educators, thus posing new challenges along with opportunities. For example, YouTube provides access to a plethora of training resources for viewers across all phases of a surgical career with the benefit of accessibility and multilevel community interaction. However, it is primarily a social media platform with a commercial intent and uploaded content does not undergo a peer-review process and has not validated quality indicators, undermining its quality. Many authors report poor video quality, inaccurate information, incomprehensible or lack of audio, and absence of background patient information [318]. The International Association of Student Surgical Societies (IASSS) has initiated a multicenter collaboration to formulate consensus statements and guidelines for open, laparoscopic, and robotic surgery videos on YouTube to ensure a minimum standard in surgical videos uploaded.

Acute care surgery has a strong impact on healthcare utilization and in-hospital cost [11–15, 107]. The cost-effectiveness of the laparoscopic approach to the most common general surgery emergencies and abdominal trauma patients is demonstrated by some studies, especially in high-income countries [48–50, 53, 245, 319, 320]. Consequently, efforts to increase the utilization of laparoscopy for emergency patients could yield immediate improvements to outcomes at both patient and system level, with reduced cost. Concerns regarding the costs of laparoscopic equipment or devices and lack of logistics such as operating room availability and trained personnel could be barriers towards the adoption of emergency laparoscopy in low-resource settings [246]. However, in this regard, some positive data related to its use were published [247] and the specific advantages of MIS such as lower surgical site infections and earlier return to work are of great benefit, especially for patients in low- and middle-income countries (LMICs). Moreover, in many low-income countries, it is difficult to promote novelties in surgery among local surgeons, due to cultural and social barriers [321, 322]. Social and economic change and partnership between manufacturers and health ministries might be the main drivers for a cost-effective healthcare in LMICs [323].

## Conclusion

The current literature review revealed several benefits of the laparoscopic approach in hemodynamically stable patients undergoing emergency abdominal and trauma surgery. The fundamental prerequisite for a safe laparoscopic approach appeared to be the selection of a stable patient, in which the laparoscopic approach was found to be feasible, reliable and effective as a therapeutic tool or helpful to guide further management steps. The inherent benefits of minimally-invasive approach to patients may exist even if conversion is eventually needed and performed. This suggests that there is an opportunity to improve the clinical trajectory for the acute care surgery and trauma patient.

The international WSES expert panel suggests laparoscopy as the first approach for stable patients undergoing emergency abdominal surgery for general surgery emergencies and abdominal trauma.

Appropriate patient selection, surgeon experience and MIS training, remain crucial factors to increase the adoption of laparoscopic approach in emergency general surgery and abdominal trauma patients, in order to improve the quality of care and reduce health care utilization rates as well as in-hospital costs.

## Data Availability

There are no individual author data that reach the criteria for availability.

## Abbreviations

AA: Acute appendicitis
ACC: Acute calculus cholecystitis
AMI: Acute mesenteric ischemia
AP: Acute pancreatitis
ASBO: Adhesive small bowel obstruction
CI: 95% Confidence interval
DCS: Damage control surgery
ICP: Iatrogenic colonoscopy perforation
IAA: Intra-abdominal abscesses
ICG: Indocyanine green
ICP: Intracranial pressure
ICU: Intensive care unit
ICU-LOS: Intensive care unit length of stay
LA: Laparoscopic appendectomy
LAMS: Lumen apposing metal stent
LOS: Length of stay
MIS: Minimally invasive surgery
NOM: Non-operative management
NOMI: Non-occlusive mesenteric ischemia
NELA: National emergency laparotomy audit
OA: Open appendectomy
OLCC: Obstructed left-side colon cancer
OR: Odds ratio 95%
PT-GBD: Percutaneous gallbladder drainage
PPU: Perforated peptic ulcer
RCTs: Randomized controlled trials
SSI: Surgical site infections
TG: Tokyo guidelines
TEP: Totally extraperitoneal
TAPP: Transabdominal preperitoneal
EUS-GBD: Endoscopic ultrasound guided gallbladder drainage
VARD: Video- assisted retroperitoneal debridement
WSES: World society of emergency surgery
